# Identification of priority areas for surveillance of cutaneous leishmaniasis using spatial analysis approaches in Southeastern Brazil

**DOI:** 10.1186/s12879-019-3940-4

**Published:** 2019-04-11

**Authors:** Diogo Tavares Cardoso, Dayane Costa de Souza, Vanessa Normandio de Castro, Stefan Michael Geiger, David Soeiro Barbosa

**Affiliations:** Departamento de Parasitologia, Instituto de Ciências Biológicas, Universidade Federal de Minas Gerais, Av. Pres. Antônio Carlos, 6627 - Pampulha, Belo Horizonte, MG 31270-901 Brazil

**Keywords:** Cutaneous leishmaniasis, Spatial data analysis, Moran’s global index, Local indicators of spatial association, Minas Gerais, Brazil

## Abstract

**Background:**

Cutaneous leishmaniasis (CL) is an important public health problem in Brazil and in several tropical regions of the world. In the Americas, Brazil is the country with the highest number of registered cases. In Brazil, the state of Minas Gerais has the highest number of cases in the southeastern region. In the present study, we used spatial analysis in the State of Minas Gerais to identify municipalities of priority during a nine-year period (2007–2015), which might be used to guide surveillance and control measures.

**Methods:**

An ecological study with spatial analysis of autochthonous cases of CL was performed in the state of Minas Gerais between 2007 and 2015. We calculated incidence rates, used Empirical Bayesian smoothing for each municipality, and divided the analyses into three-year intervals. In order to analyze the existence of spatial autocorrelation, and to define priority areas, Moran’s Global Index and Local Indicators of Spatial Association (LISA) were used.

**Results:**

The mean incidence rate for the entire state was 6.1/100,000 inhabitants. For Minas Gerais, analysis of CL cases over time revealed a successive increase of indicated mesoregions with high priority municipalities. Eight of the designated mesoregions contained municipalities classified as high priority areas in any of the three evaluated trienniums, and four mesoregions had high priority municipalities throughout the entire investigation.

**Conclusions:**

Within the southeastern region of Brazil, Minas Gerais State stands out, with highest CL incidence rates. Using spatial analysis, we identified an increasing numbers of cases in the municipalities classified as high priority areas in different mesoregions of the state. This information might be of value to direct surveillance and control measures against CL and to understand the dynamics of the expansion of CL in Minas Gerais. Similar approaches might be used to map CL in other regions throughout Brazil, or in any other country, where national notification and control programs exist.

## Background

Leishmaniasis is an important worldwide public health problem, being the second most important vector-borne disease caused by protozoan parasites, after malaria. Cutaneous leishmaniasis (CL) in humans is caused by protozoan species of the gender *Leishmania* spp*.,* which are endemic in 98 countries of the world, with estimates of about 0.7 to 1.2 million new infections per year. Roughly 75% of the notified cases originate in the following countries: Afghanistan, Algeria, Iran, Syria, Sudan, Costa Rica, Peru, Colombia and Brazil [1, 2]. In the New World, the majority of cases are reported in Brazil. [3].

In the 1980s, Brazil had an annual increase of approximately 3000 new clinical CL cases. This number further rose to 38,000 new annual cases from the year 2000 onwards [4]. Between 2007 and 2015, the disease was diagnosed in all Brazilian states, with the highest numbers of new cases being registered in the states of Pará (32,201), Bahia (27,906), and Mato Grosso (23,099). In this period, the State of Minas Gerais occupied the sixth position in the ranking of most-affected states, with 11,120 cases. Thus, in the southeastern region of Brazil, Minas Gerais had a much higher number of cases than, for example, the States of São Paulo, Espírito Santo, or Rio de Janeiro, with 2910, 907, or 525 new cases, respectively [4].

Historically, CL has been characterized in Brazil as a zoonosis, endemic mainly in rural areas, affecting wild mammals and occasionally humans, especially when the latter is exposed to the sylvatic transmission cycle of CL. Such situations frequently occur during the construction of highways or railroads [5]. Today, CL infections and active transmission are reported from nearly deforested regions, and from peri-urban and urban areas [6].

In order to supervise and control CL, a surveillance program has been launched by the Brazilian Ministry of Health, with one of the objectives being the identification and monitoring of territorial units of epidemiological significance for control measures [4]. In this respect, Geographic Information Systems (GIS) are a powerful tool to gather information regarding the epidemiology of infectious diseases. GIS can be used to identify priority areas and guide the implementation of surveillance programs and interventions for control. As such, spatial analysis has been widely used, and has allowed, for example, the identification and characterization of areas with high CL incidence and with elevated morbidity [7–11]. In Minas Gerais, previous studies drew attention, in particular to areas with high incidence rates, such as Jequitinhonha [6, 12, 13] or the North of Minas Gerais [14], in order to understand epidemiological aspects of CL in a restricted region of the state.

In the present study, we applied spatial analysis of CL cases from the entire state of Minas Gerais during the period from 2007 to 2015, searched for regions with agglomeration of the disease, and municipalities with high priority areas for surveillance, and investigated how these regions behave over time. The application of GIS was used to describe patterns of spatial and temporal distribution, and aimed to identify municipalities of priority, which might help to guide surveillance and control measures in the future.

## Methods

### Study area and epidemiological design

**Fig. 1:**
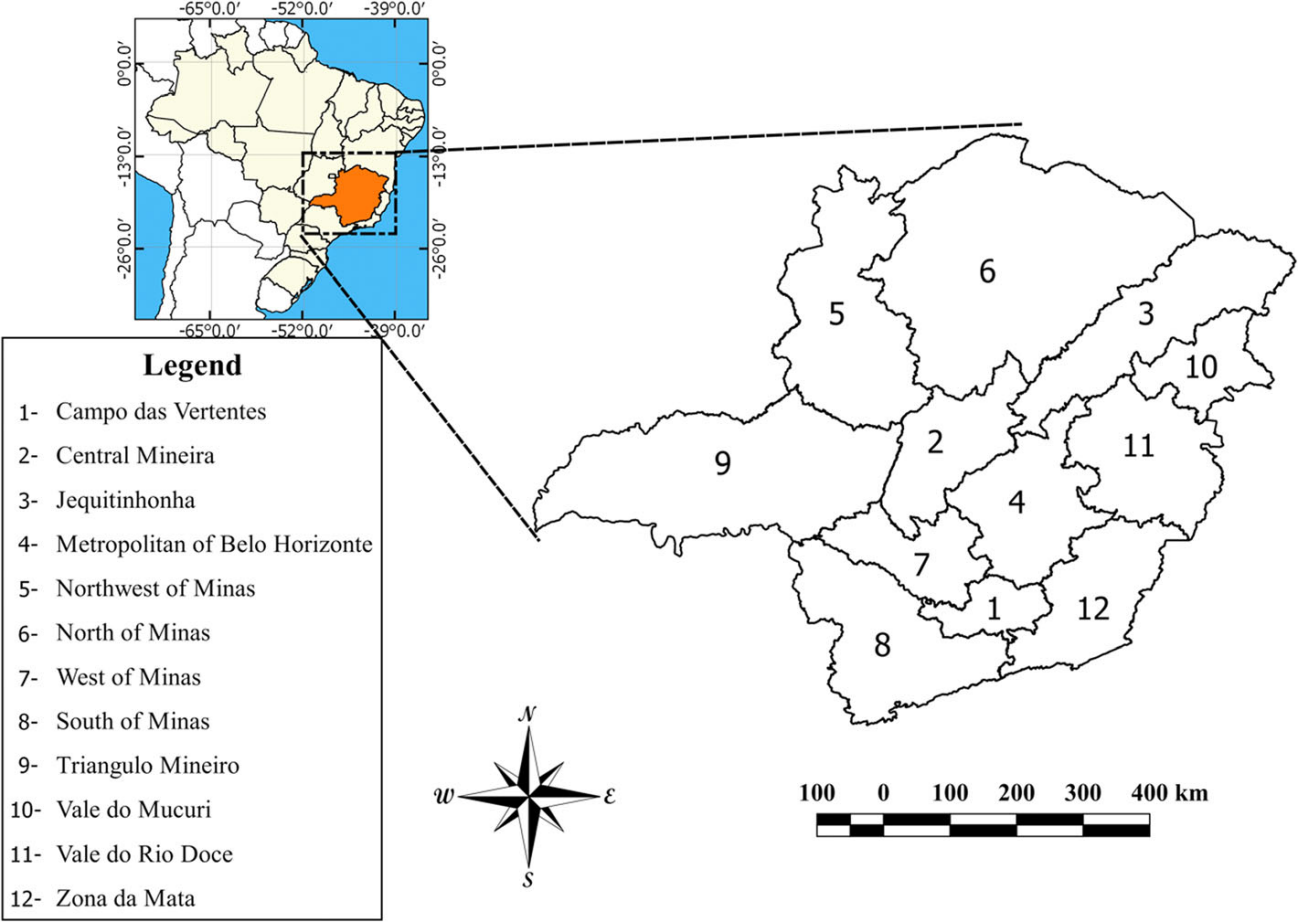
Map of the study area of Minas Gerais and its municipalities and territorial divisions in mesoregions. Localization of the state of Minas Gerais in Brazil (small map) and its division into twelve mesoregions with its respective denominations (zoom)

The State of Minas Gerais is one of 27 federative units of Brazil and it is located in the Southeast region, between the latitudes 14°13′58" and 22°54’00" south and the longitudes 39°51′32"and 51°02′35" west (Fig. 1). It is the fourth most extensive state, with a territorial area of 586 million km^2^, which represents approximately 7% of the entire Brazilian territory. Minas Gerais is subdivided into 853 municipalities, is the state with the highest number of municipalities, and an estimated population in 2018 of about 21 million inhabitants [15].

We performed an ecological study, based on spatial analysis techniques, and using secondary data of confirmed autochthonous CL cases. In order to do so, the database of the national surveillance system for notifiable diseases of the Brazilian Ministry of Health was accessed (SINAN - *Sistema de Informação de Agravos de Notificação*). Data from between period of 2007 to 2015 were used, where the analytical units were the 853 municipalities of Minas Gerais.

### Incidence rates and spatial analysis

Cases of CL were aggregated according to each municipality (analytical unit), and CL incidence rates were calculated for each of the state mesoregions (i.e., geopolitical subdivisions that encompass several municipalities with economic and social similarities) and municipalities. Population data for each municipality were obtained from the Brazilian Institute of Geography and Statistics (*Instituto Brasileiro de Geografia e Estatística – IBGE),* based on the National Population Census performed in 2010. The incidence rates were combined in three-year intervals and divided into first (2007–2009), second (2010–2012), and third trienniums (2013–2015).

We created a contiguity spatial weight matrix for each analytical unit’s level. The terms used in the weight matrix were based on first order neighborhood criteria, which define neighbors as those areas with shared borders and vertices (q*ueen*)*.* To reduce random fluctuation, and to facilitate subsequent spatial data analysis, we calculated CL incidence rates for each of the geographical analytical units and re-estimated them using a Spatial Empirical Bayesian smoothing approach For the Bayesian calculations, we used the software GeoDa version 1.10 (ASU, GeoDa Center for Geospatial Analysis and Computation, Arizona, USA).

In order to check whether the incidence rates in neighboring areas occurred randomly or followed some pattern or spatial dependence, we performed autocorrelation between CL incidence rates, and calculated Moran’s Global Index. The incidence rates of CL per 100,000 inhabitants in each triennium and analytical unit were used, followed by the application of Moran’s *I* statistic. The continuous Moran’s Global index value can vary from − 1.0, indicating an inverse correlation (dispersed), to + 1.0, indicating a direct correlation (clustered). Values of zero or close to zero are supposed to indicate random distribution [16].

In a second step, Local Indicators of Spatial Association (LISA) were determined for the identification of local spatial clusters in each triennium. LISA analysis divides the value of Moran’s Global index, reflecting the value of each unit of analysis, and indicates whether there are associations with neighbors, and the presence or absence of outliers [17].

The LISA values were presented in four quadrants: high priority area with units added in Quadrant 1 – high/high (positive values, positive means); lower priority areas with units added in Quadrant 2 – low/low (negative values, negative means); intermediate priority areas with aggregate units in Quadrant 3 – high/low (positive values, negative means) and Quadrant 4 – low/high (negative values, negative means). The first two categories represented concordance areas, and the last two transition areas [17]. LISA significance maps for CL indicators were constructed for the analytical units, and the significance level for spatial autocorrelation was set to *P* ≤ 0.05. Digital maps were obtained from the IBGE Cartographic data of the territory of Minas Gerais-Brazil was taken from the Brazilian Institute of Geography and Statistics (IBGE) website in shapefile format. This spatial data has public domain. The figures (maps) were made by the authors considering this open geographical source [15]. Analyses of Moran’s Global Index and LISA were performed using GeoDa Software version 1.10, and maps were constructed using the QGIS 2.18 software.

### Ethical considerations

This study was based on secondary data, and all presented information is in the public domain. None of the variables or data used in this study allowed the identification of individuals. Thus, approval of the study by an Ethical Review Board was not necessary.

## Results

**Fig. 2:**
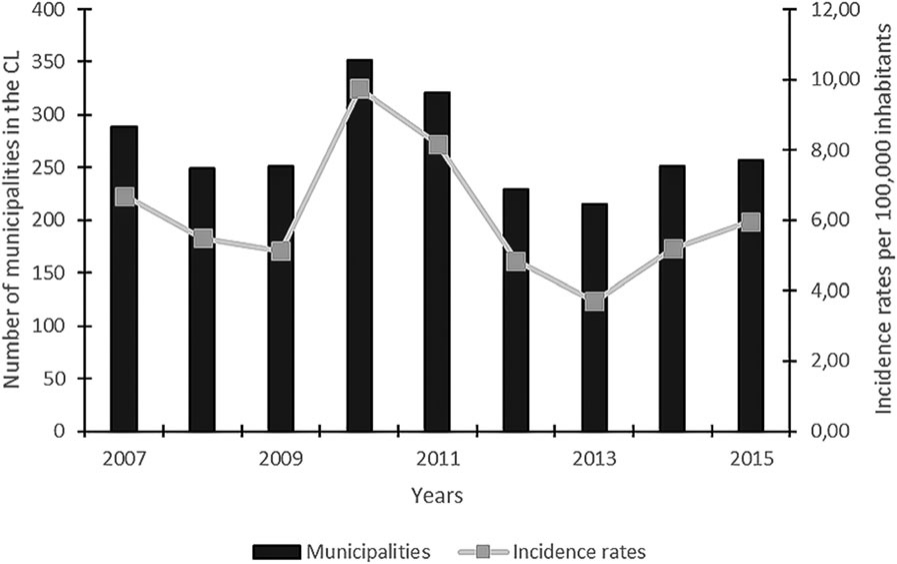
Incidence rates of cutaneous leishmaniasis and number of municipalities of cutaneous leishmaniasis, 2007–2015. Temporal distribution of cutaneous leishmaniasis (CL) in the state of Minas Gerais during the time period from 2007 to 2015. Indicated are the incidence rate (line) and the number of municipalities with confirmed CL cases (black bars) in function of time

In Minas Gerais, the average CL incidence rate was 6.1/100,000 inhabitants during the study period from 2007 to 2015. 72.9% (622/853) of the municipalities presented at least one case of CL during the analyzed period. The overall incidence rates oscillated between 9.8/100,000 in 2010, to 3.7/100,000 in 2013. The number of municipalities with confirmed CL cases was highest in 2010 (*n* = 352) and lowest in 2013 (*n* = 215). The temporal fluctuation profile of municipalities with CL-positive cases followed the fluctuation in overall incidence rates in the municipalities of Minas Gerais, with maximum values in the years 2010 and 2011 (Fig. 2). Even with the peak in the year 2010, there was a decrease in the incidence rate of CL.

**Fig. 3:**
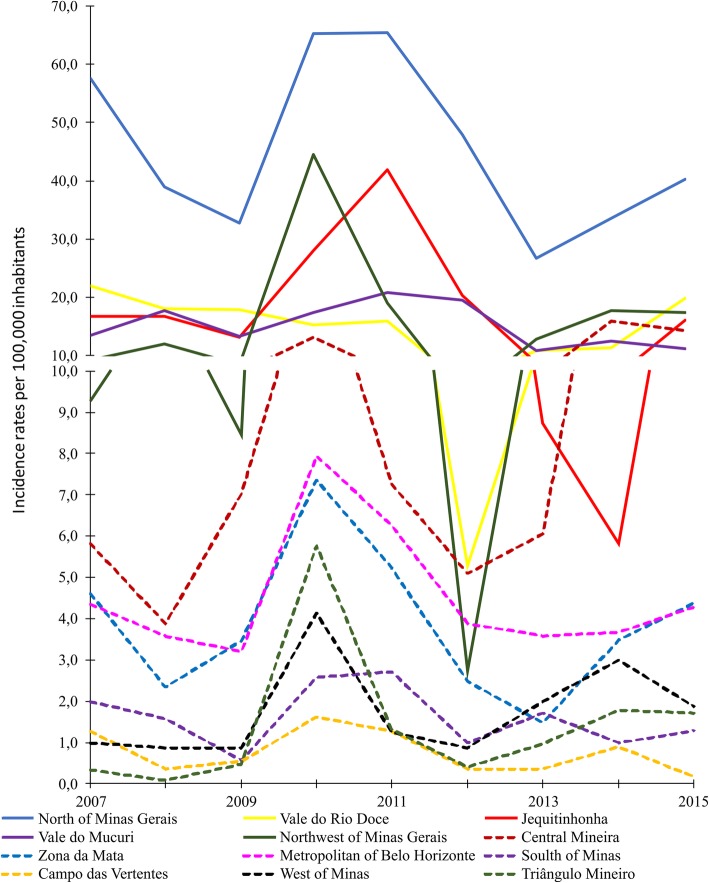
Incidence rates of cutaneous leishmaniasis in the mesoregions of Minas Gerais State, 2007–1015. Incidence rate of cutaneous leishmaniasis (CL) in the twelve mesoregions of Minas Gerais, during the time period from 2007 to 2015

Almost 81% of all CL infections were concentrated in five mesoregions, namely North of Minas (34.8%), Vale do Rio Doce (15.6%), Jequitinhonha (11.8%), Metropolitan of Belo Horizonte (11.7%), and Zona da Mata (7.0%). Furthermore, out of all municipalities, Januária, located in Northern Minas Gerais, was the one with the highest number of registered CL cases (761), nearly 50% more cases than Montes Claros, the municipality that ranged second (444). Again, North of Minas, Vale do Rio Doce, and Jequitinhonha were mesoregions with the highest incidence rates. Interestingly, in the Northwest of Minas, a considerable increase in the incidence rates was observed in 2010 (44.5/100,000 inhabitants), followed by a reduction in 2012 (2.7/100,000 inhabitants), and newly increased values during the last three years of the evaluation (Fig. 3).

**Fig. 4:**
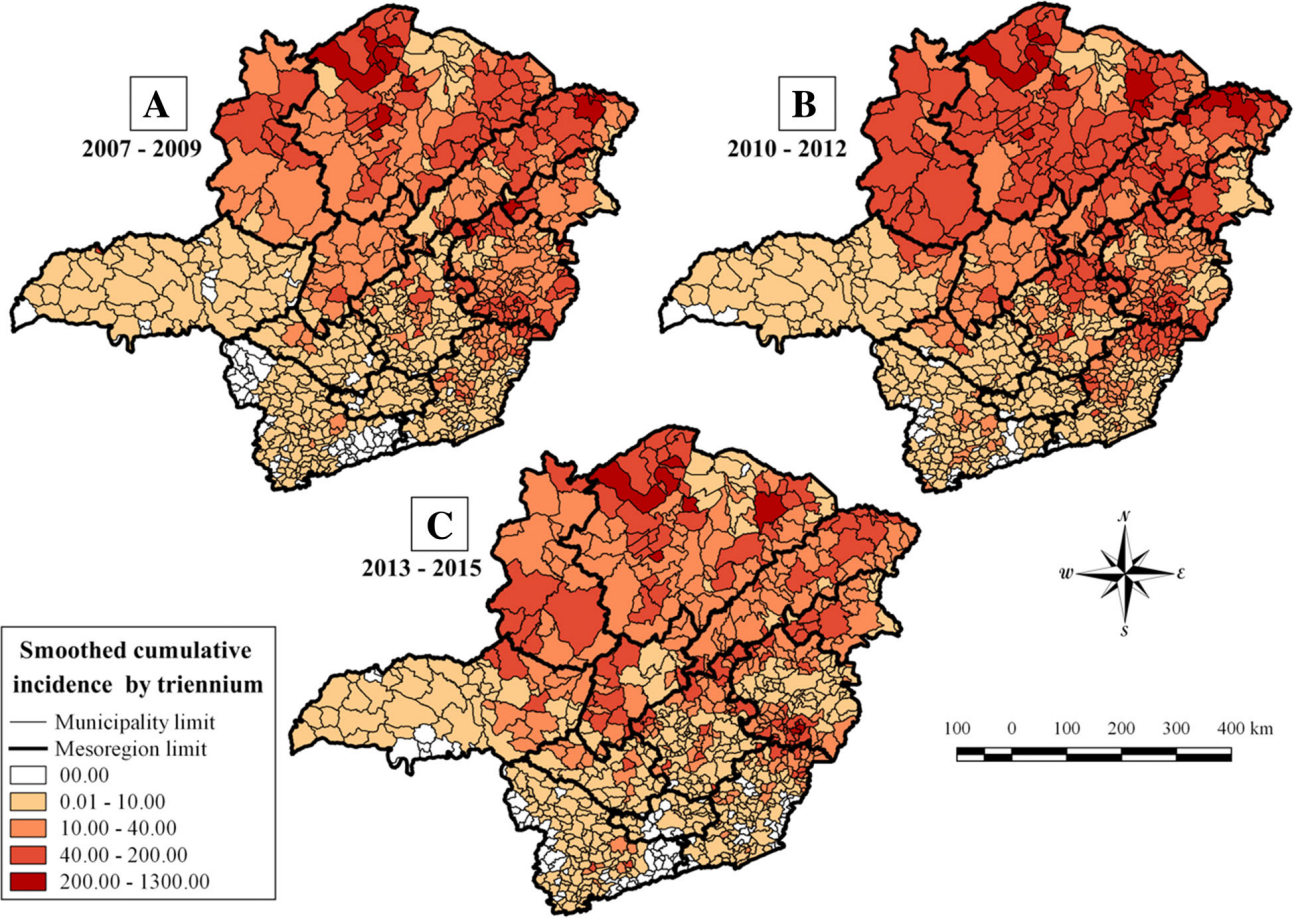
Mapping of incidence rates of cutaneous leishmaniasis in Minas Gerais during the years from 2007 to 2009. Smoothed cumulative incidence rates of cutaneous leishmaniasis (CL) per 100,000 habitants in the municipalities of Minas Gerais state were shown during the time period from 2007 to 2015 and were indicated in three-year intervals from 2007 to 2009 (**a**), 2010–2012 (**b**) and from 2013 to 2015 (**c**)

The highest incidence rates were concentrated in the mesoregions North of Minas, Northwest of Minas, Jequitinhonha, Central Mineira, Vale do Mucuri and Vale do Rio Doce. All of these mesoregions are located in the upper half of the state, and had the majority of their municipalities indicated with smoothing incidence rates higher than 10.0/100,000 inhabitants (Fig. 4). The incidence rates in the Triângulo Mineiro region had low values in the first triennium, with only one municipality with more than 10 cases per 100,000 inhabitants. However, the number of municipalities that were indicated with incidence rates higher than 10.0/100,000 rose to 14 in the third triennium. These municipalities were located at the division of the mesoregions Northwest of Minas and Central of Minas, which were much more affected by CL throughout the study period (Fig. 4a and c).

**Fig. 5:**
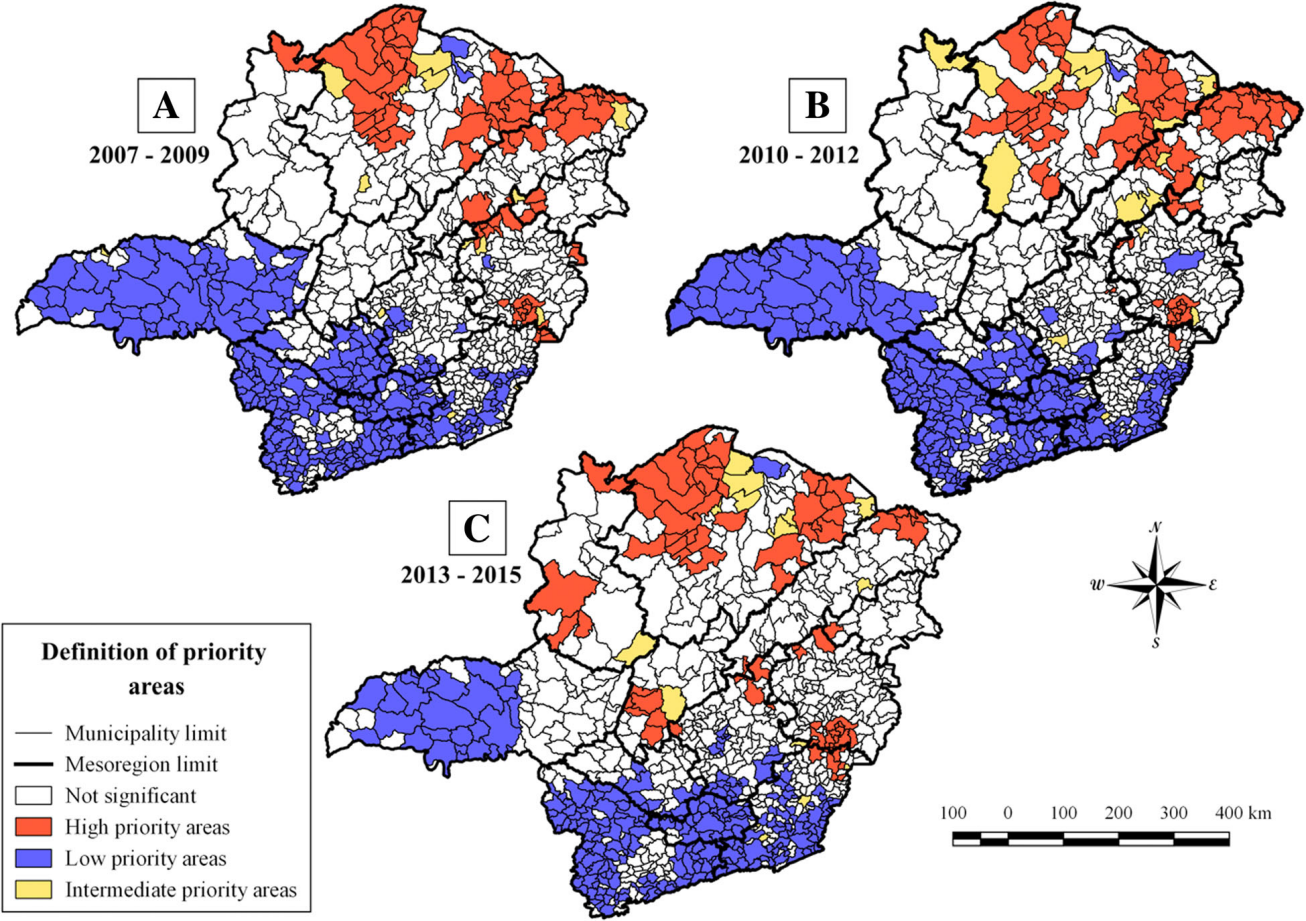
Identification of high priority areas for cutaneous leishmaniasis on the municipality level in Minas Gerais. Identification of priority areas for control and surveillance of cutaneous leishmaniasis (CL) in the municipalities of Minas Gerais state, during the time period from 2007 to2015. High (red), intermediate (yellow) and low priority areas (blue) are indicated for the trienniums from 2007 to 2009 (**a**), 2010–2012 (**b**) and from 2013 to 2015 (**c**)

The Global Moran’s Index revealed a statistically significant and positive spatial autocorrelation regarding the registered incidence of CL, and thus indicated spatial dependence for Minas Gerais during the years from 2007 to 2015. In addition, there was little variation in the Global Moran’s *I* statistic calculated for the three-time periods (Table 1). For the entire state of Minas Gerais, the number of municipalities classified as high priority areas by LISA decreased from 75 for the first triennium to 71 for the third triennium (Table 2). During the first triennium, the mesoregions North of Minas (36), Vale do Rio Doce (17) and Jequitinhonha (13) were most affected, and concentrated 88% of the municipalities that were classified as high priority. In contrast to that, the mesoregions Vale do Mucuri and Zona da Mata had the lowest number of municipalities with high priority areas (Table 3 and Fig. 5a).

**Table 1:** Global Moran’s Index statistics for cutaneous leishmaniasis in Minas Gerais, Brazil

Time period	Global Moran’s Index	*P* value
Triennium I (2007–2009)	0.3771	< 0.01
Triennium II (2010–2012)	0.3771	< 0.01
Triennium III (2013–2015)	0.3319	< 0.01

Calculation of the Moran’s Global Index for the State of Minas Gerais, based on the occurrence of human CL cases during three trienniums from 2007 to 2009, 2010–2012, and from 2013 to 2015

**Table 2:** Number of priority municipalities identified according to LISA

Time period	High priority areas	Low priority areas	Intermediate priority areas	Not significant
Triennium I (2007–2009)	75	279	14	485
Triennium II (2010–2012)	74	269	17	493
Triennium III (2013–2015)	71	254	15	513

Number of high, low, and intermediate priority municipalities for surveillance and control of cutaneous leishmaniasis (CL), according to Local Indication of Spatial Association (LISA) values in the State of Minas Gerais in relation to the investigated periods from 2007 to 2015

**Table 3:** Number of high priority municipalities for cutaneous leishmaniasis according to different mesoregions of Minas Gerais

Mesoregion	Triennium I (2007–2009)	Triennium II (2010–2012)	Triennium III (2013–2015)
North of Minas Gerais	36	39	30
Vale do Rio Doce	17	11	15
Jequitinhonha	13	20	6
Vale do Mucuri	4	2	1
Zona da Mata	4	1	6
Northwest of Minas Gerais	1	0	4
Metropolitan of Belo Horizonte	0	1	3
Central Mineira	0	0	6
Total	75	74	71

The numbers of high priority municipalities were split into three trienniums from 2007 to 2015 and separated according to different mesoregions of Minas Gerais

The mesoregion North of Minas had the highest number of municipalities classified as high priority areas. When compared with the first triennium, high priority areas in the second triennium in the North of Minas showed an increase of 8.3% (36 to 39 municipalities), whereas in the third triennium a reduction of 23.1% was observed, when compared with the second triennium. Nevertheless, North of Minas continued as the mesoregion with the highest number of municipalities classified as high priority throughout the three evaluated trienniums (Table 3). The mesoregion Jequitinhonha had the highest temporal fluctuations in the number of municipalities indicated as high priority areas. Notably, from the second to the third triennium, a considerable reduction in high priority areas of 70.0% was observed (20 to 6 municipalities) (Table 3 and Fig. 5b, c).

The four mesoregions Zona da Mata, Northwest of Minas Gerais, Metropolitan of Belo Horizonte, and Central Mineira were identified with low but increasing numbers of municipalities classified as high priority during the study period. In the case of the mesoregion Metropolitan of Belo Horizonte, the high priority areas, which appeared during the third triennium, were located near the Vale do Rio Doce mesoregion, and most probably spread from there to this more central region. Interestingly, the few high priority areas of the mesoregions Central Mineira and Northwest of Minas Gerais, were relatively isolated but clustered, and appeared only during the last triennium from 2013 to 2015 (Table 3 and Fig. 5).

**Fig. 6:**
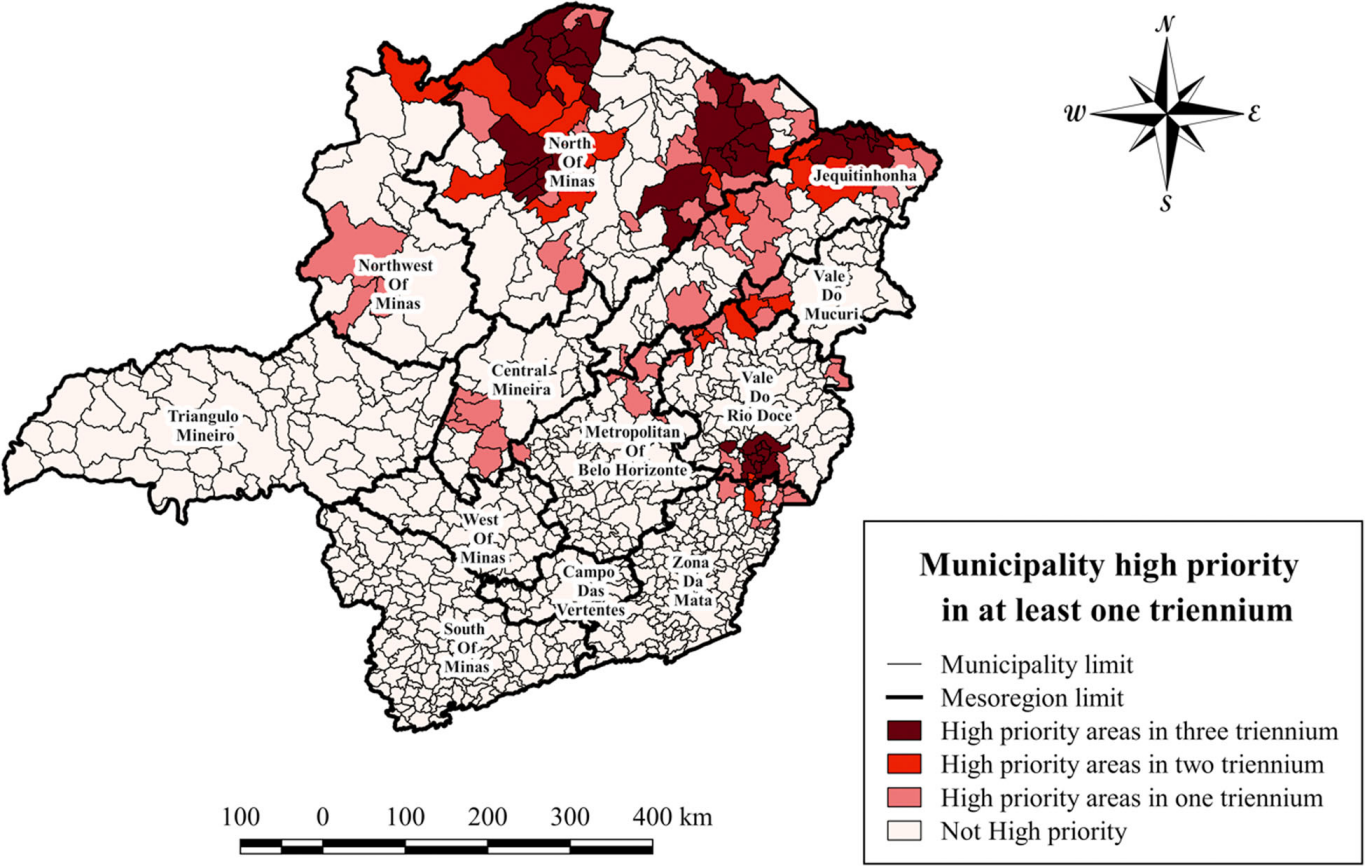
Mapping of high priority municipalities for cutaneous leishmaniasis inMinas Gerais. Identification of priority areas for cutaneous leishmaniasis (CL) on the municipality basis and combined for the three trienniums from 2007 to 2015. Brown areas: municipalities classified as high priority in all three years; red areas: municipalities classified as high priority in two trienniums; pink areas: municipalities classified as high priority in only one triennium; white areas: municipalities that were not classified as high priority

In total, 124 municipalities were classified as high priority areas. Only 36 of these municipalities were classified as high priority during all three trienniums, with all of them being concentrated in the most affected mesoregions, namely North of Minas (24), Jequitinhonha (5) and Vale do Rio Doce (7) (Fig. 6). Additionally, 88 municipalities were classified as high priority during at least one triennium: North of Minas (24); Jequitinhonha (19); Vale do Rio Doce (16); Central Mineira (6); Vale do Mucuri (5); Northwest of Minas (4) and Metropolitan of Belo Horizonte (4).

During the investigated period, we were able to identify municipalities with intermediate priority in the first (14), second (17), and third triennium (15) (Figs. 5). Most of these were located next to high priority areas and, most probably, were influenced by them and might become high priority areas in the near future. On the other hand, the number of municipalities with low priority diminished from 279 in the first to 254 in the third triennium, indicating a decline of these areas and expansion of intermediate areas (Table 2; Figs. 5). In this respect, in particular, the mesoregions Triângulo Mineiro, South of Minas, and Campo das Vertentes, will require attention in the future, in order to try to prevent the expansion of high priority municipalities with the subsequent need for special surveillance and control of CL.

## Discussion

Since the 1980s, incidence rates for CL in Brazil showed a steady annual increase, and an important spatial diffusion to almost all of the 27 Brazilian member states, turning this protozoan infection into a problem for public health services. In terms of total numbers, confirmed CL cases rose from 3000 in the 1980s to 20,000 newly registered annual human cases in 2014 [4]. In Latin America, taking advantage of GIS utilities and indicating most-affected regions, CL was reported in fourteen countries between 2001 and 2011, with an increase of 30% in reported cases [3]. In the southeastern region of Brazil, the State of Minas Gerais is of importance, not only because of its geographical extension, but also because of more than 1000 new CL cases per year, which is much more than in neighboring states, and even in comparison to whole Latin American countries, such as Costa Rica, Honduras and México [3]. Thus, within the national context, this region turned into an endemic area of special attention for the Brazilian Ministry of Health [4]. Here, during the period of our study, we were able to observe an overall tendency for a reduction in CL cases in the State of Minas Gerais. However, by using GIS and smaller functional units, priority areas with increasing incidence rates were detected.

In the present study, we evaluated the incidence rates of CL for each of the more than 800 municipalities of Minas Gerais, and designed risk maps for the period from 2007 to 2015. We tried to understand spatial and temporal changes involving the disease, and identified spatial clusters within this state, using GIS tools. Similar studies have already been published for CL in other endemic countries, such as Iran and Morocco, with the aim to identify priority areas [7, 8] or to associate environmental factors with increased incidence [18]. In Brazil, some studies using similar methods were conducted to search for priority areas for the control of malaria [19] or for visceral and CL [10, 11, 20]. Also in Brazil, other studies used similar tools to identify high priority or risk areas for mortality caused by Chagas disease [21] or by visceral leishmaniasis [22].

In Brazil, *L. braziliensis*, *L. amazonensis*, and *L. guyanensis* have been the most frequent causes of CL in humans, and the disease has been linked to sylvatic transmission cycles of the parasite in its natural setting, and when humans come into contact with these areas, for example, often during the construction of highways or railroads [5]. Increased incidence rates in urban environments were shown to be mainly due to changes to the natural environment caused by human activity, and occurred in nearly deforested urban or peri-urban settings, as for instance documented in Minas Gerais [6] and Parana states [23].

For Minas Gerais state, the number of cases resulted in an average incidence rate of 6.1/100,000 inhabitants, which is below the national average of 14.7/100,000 inhabitants [4]. When incidence rates were calculated for the different mesoregions, peak rates were observed in the year 2010. In this respect, a highly elevated incidence rate of CL was, in particular, observed in the mesoregion Northwest of Minas, i.e. five times greater when compared with the year 2009. On the other hand, since a peak in incidence rates occurred in almost all of the mesoregions in 2010, this might indicate a common, but yet unknown, environmental influence. Other studies conducted in South America, showed that CL was associated with climate changes and their impact on the environment [24]. In 2009–2010, there was an occurrence of the El Niño-Southern Oscillation, and other studies have associated temporal fluctuations and high incidence rates of CL cases subsequent to this phenomenon [25, 26]. Increased CL incidence is supposed to occur due to an increase in sandfly populations during and shortly after El Niño events [26]. According to Brazilian national weather data, there was an increased amount of precipitation from 2007 to 2009, above the historic average, something that did not occur in 2010 [27]. Also, above-average precipitation was again observed in 2013 [27]; although with no major increase in CL incidence rates in Minas Gerais. Nevertheless, this phenomenon, and the effects on local climatic factors, might have played a role in the epidemiology and peak incidence rates of CL in Minas Gerais in 2010, as also shown by others during earlier studies [28].

In the present study, the majority of CL cases were clustered in five mesoregions: North of Minas, Vale do Rio Doce, Jequitinhonha, Metropolitan of Belo Horizonte, and Zona da Mata. The existence of spatial clusters of municipalities classified as high priority areas may indicate a predisposition of spatial distribution, which, most probably, might be driven by environmental factors [18, 29]. In other studies, it was reported that the existence of such spatial clustering facilitated targeted interventions and rendered them more effective [30, 31]. In particular, temperature and humidity [18], or the presence of desert areas [32], were environmental factors that have contributed to spatial clustering in other regions of the world.

Here, we identified out 8 mesoregions of importance for CL surveillance and control, and which contained a varying number of high-priority municipalities with elevated incidence rates and considerable degrees of clustering within or between mesoregions. Some former, but also very informative, studies on the epidemiology of CL in Brazil calculated the cases of CL per km^2^ and identified poles or circuits of aggregated human cases in more restricted urban or rural areas [33, 34]. The Brazilian Ministry of Health adopted this method to identify areas of concentration of CL cases [4]. Using this former method, 31 production circuits of CL were identified in Brazil, of which the state of Minas Gerais contained 3 production circuits for the time period from 2007 to 2013, being located in the mesoregions Metropolitan of Belo Horizonte, Vale do Rio Doce and North of Minas [4]. In comparison, in the present study, LISA was applied and it was possible to identify clustered high-priority areas in eight mesoregions, instead of only three in the former study, but including the formerly cited mesoregions, as well. In general, exploratory techniques, such as the calculation of Global Moran’s Index in association with LISA, are very useful in identifying priority areas for the implementation of public policies related to disease monitoring and control [35], and might be superior and more detailed than the indication of priority circuits, as proposed by others [4, 34].

The monitoring of municipalities classified as high priority for surveillance is important because these municipalities may influence their neighbors, and spreading of reservoir hosts or vectors for CL may take place, as reported, for example, for the state of Rio de Janeiro [36]. One important factor for the increase of incidence rates and expansion of high priority areas for CL could be migration of reservoir hosts to the close vicinity of housing, as was already reported for Minas Gerais and Rio de Janeiro [14, 36]. Importantly, one of those studies indicated that the expansion of CL between neighboring municipalities or even smaller geographical units occurred independently from deforestation or human migration [36]. Even though that study used a different scale for the distribution of CL [36], we should not exclude that the latter hypothesis for the expansion of CL might also hold true for some municipalities in Minas Gerais. In this respect, small cosmopolitan rodents were shown to have a certain affinity for humans, and their food resources were of great importance for the maintenance of both the wild and the peri-domestic cycles of CL [37]. Due to their mobility of these small rodents, process of disease spread may be related to them due to the circumstance the they move between regions and being thus able to transport it to nearby regions [34, 36–39].

In Minas Gerais, several wild animal species were reported to be infected with *L. braziliensis*. Interestingly, the main mammalian species encountered were *Rattus rattus* and *Mus musculus*, cosmopolitan rodents with a history of living in close proximity to humans [38]. Other studies also pointed out the importance of rodents and small wild mammals as natural hosts for different *Leishmania* species, and for their roles in the maintenance of the CL parasite lifecycle and transmission [14, 39]. In the southeastern region of Brazil, these reservoir hosts were mainly infected with *L. braziliensis,* which also was incriminated as the main cause of CL in humans in this region [13, 14, 38].

Another important factor, which contributes to the increase in CL cases, is the presence of sandfly vectors. Several studies in different regions of the state have identified the presence of appropriate sandfly species [40–45]. With the presence of the vector in several areas, the infectious cycle can easily be established and lead to increased human CL incidence rates. Also, the observed urbanization of sandflies is an important factor in the expansion of the disease, as was reported for Minas Gerais [42]. However, in the present retrospective study, it was beyond our scope to include any further data on the presence of sandfly vectors, reservoir hosts, or other environmental factors for the different mesoregions of Minas Gerais investigated, or to check for their association with human incidence rates. The incidence rate had a tendency to decrease over the period of this study, and the prioritization of areas identified through these studies may have enhanced the tendency to decrease the incidence rate of CL.

Since this study was based on secondary data, it has certain limitations in relation to the availability and use of secondary data. Problems in endemic areas may have led to incomplete notifications, missing, or unknown information surrounding CL cases. Also, the contribution and weight of socio-economic and/or environmental factors involved in the spreading of CL in the state of Minas Gerais were not investigated here, but might be addressed in future studies.

Despite these limitations, the data were sufficiently robust to present statistically significant information regarding the increase in incidence rates of CL, and for the expansion of priority municipalities for surveillance in Minas Gerais.

## Conclusions

Minas Gerais presents with high incidence rates of CL, with most cases concentrated in the southeastern region. In our study, 124 municipalities in Minas Gerais were classified as high priority in at least one triennium, of which 36 municipalities were classified as high priority during the whole study period and which were located in the mesoregions North of Minas, Jequitinhonha and Vale do Rio Doce. These 36 municipalities deserve special attention for control, since they can influence neighboring areas, and might contribute to the expansion of CL within and beyond the state.

Our calculations of Global Moran’s Index and LISA risk mapping allowed the identification and quantification of stabile and expanding areas of CL cases over time, as well as the identification of regions that share similar spatial patterns. The results reported herein may assist and guide the implementation of disease control measures in the state of Minas Gerais. In addition, the presented approach to detect and describe regions of high priority for CL in this vast geographic area might be applied to other regions, or diseases, in Brazil or beyond.

## Abbreviations

CL: Cutaneous leishmaniasis
CPTEC: Weather Prevision Center and Climate Studies (Centro de Previsão de Tempo e Estudos Climáticos)
GIS: Geographic Information Systems
IBGE: Brazilian Institute of Geography and Statistics (Instituto Brasileiro de Geografia e Estatística)
INPE: National Institute of Space Research (Instituto Nacional de Pesquisas Espaciais)
Km^2^: Square Kilometer
LISA: Local Indicators of Spatial Association
SINAN: Brazilian Health Notification System (Sistema de Informação de Agravos de Notificação)
